# Nickel nanoparticles: a novel platform for cancer-targeted delivery and multimodal therapy

**DOI:** 10.3389/fddev.2025.1627556

**Published:** 2025-07-30

**Authors:** Fengyu Wang, Sen Tong, Xuan Ma, Huan Yang, Tianbao Zhang, Kunrong Wu, Junzi Wu

**Affiliations:** ^1^ The Key Laboratory of Microcosmic Syndrome Differentiation, Yunnan University of Chinese Medicine, Kunming, China; ^2^ Yunnan Key Laboratory of Integrated Traditional Chinese and Western Medicine for Chronic Disease in Prevention and Treatment, Yunnan University of Chinese Medicine, Kunming, China; ^3^ School of Chinese Medicine, Yunnan University of Chinese Medicine, Kunming, China

**Keywords:** nickel nanoparticles, drug delivery, photothermal therapy, magnetic hyperthermia therapy, chemodynamic therapy

## Abstract

Traditional cancer treatment methods often encounter limitations, such as poor targeting, low bioavailability, and high systemic toxicity. These challenges have led researchers to explore alternative therapeutic strategies. Nickel nanoparticles (NiNPs), owing to their distinctive physicochemical properties and tunable biocompatibility, have attracted considerable attention in cancer therapy and drug delivery applications. These nanomaterials demonstrate excellent magnetic properties, photothermal conversion capabilities, catalytic activity, and potential for multifunctionality and targeted drug delivery via surface modification. This review highlights recent advancements in the use of NiNPs for cancer treatment, emphasizing their advantages as drug carriers that enhance the bioavailability, targeting, and therapeutic efficacy of anticancer agents. Additionally, the synergistic applications of NiNPs in multimodal therapies, including magnetic hyperthermia, photothermal therapy, and chemodynamic therapy, are discussed, as well as their potential as theranostic platforms. Although nickel-based nanodelivery systems show significant promise for clinical translation, issues related to biosafety, degradation metabolism, and long-term toxicity remain and require further investigation to support their clinical application.

## 1 Introduction

Cancer has become a major global public health concern. Statistical data indicate that approximately 20 million new cancer cases occurred worldwide in 2022, resulting in around 9.7 million deaths (Dolgin, 2021; Filho et al., 2025). Although conventional chemotherapeutic agents exhibit substantial antitumor activity *in vitro*, typically less than 0.1% of the administered dose reaches the tumor site. Most of the drug is instead distributed to healthy tissues or eliminated by the reticuloendothelial system (Cornen and Vivier, 2018; Niu et al., 2025). This inefficient delivery not only reduces therapeutic efficacy but also increases the risk of toxicity to normal cells. The advancement of nanotechnology has introduced new opportunities to overcome these limitations. Nanocarriers can enhance therapeutic outcomes by improving drug bioavailability, selectivity, and efficacy in target tissues while minimizing toxicity to healthy cells (Haider et al., 2020; Lepeltier et al., 2020). Recent findings suggest that rationally designed nanocarriers can increase drug accumulation in tumors by five-to ten-fold compared to free drugs, thereby enhancing therapeutic effects and reducing systemic toxicity (Haripriyaa and Suthindhiran, 2023; Khizar et al., 2023).

Despite the promise of nanotechnology, existing metallic nanoparticles face limitations that impede clinical translation. For example, gold nanoparticles possess excellent biocompatibility but lack magnetic responsiveness and have limited catalytic activity in chemodynamic therapy (CDT) applications (Nam et al., 2021; Kesharwani et al., 2023; Aggarwal et al., 2025). Silver nanoparticles, although known for their antimicrobial properties, raise concerns regarding long-term toxicity and lack the multifunctionality required for comprehensive cancer treatment (Ferdous and Nemmar, 2020; Takáč et al., 2023). These shortcomings highlight the urgent need for novel nanomaterial platforms. In comparison to other metallic nanoparticles, nickel nanoparticles (NiNPs) offer several distinct advantages. Unlike gold nanoparticles, which primarily rely on photothermal mechanisms, NiNPs function through magnetic targeting, catalytic activity for chemodynamic therapy, and efficient photothermal conversion under NIR-II irradiation (Lei et al., 2019; Roy et al., 2023). The magnetic susceptibility of nickel allows for precise external field manipulation to achieve targeted delivery, a feature absent in precious metals such as gold and silver. In addition, nickel’s unique electronic structure enables Fenton-like catalytic reactions, generating reactive oxygen species (ROS) more effectively than iron-based systems, while offering superior biocompatibility compared to copper-based alternatives (Wu et al., 2022; Dawson et al., 2023). Moreover, compared to precious metals, such as gold and platinum, the cost-effectiveness of nickel renders NiNPs a more economically viable option for large-scale clinical applications, potentially enhancing the accessibility of advanced cancer therapies (Zhou et al., 2023).

NiNPs exhibit exceptional magnetic properties, catalytic activity, photothermal conversion efficiency, and surface modification potential, rendering them promising candidates for drug delivery systems and cancer therapeutics (Ma et al., 2022; Berhe and Gebreslassie, 2023). Their magnetic characteristics enable precise manipulation via external magnetic fields, facilitating targeted delivery to tumor sites and enhancing drug accumulation efficiency at these locations (Bouremana et al., 2022). In addition, their notable catalytic properties promote specific chemical reactions within the tumor microenvironment, generating ROS that induce apoptosis in cancer cells (Shubhra, 2023; Graham et al., 2025). Through strategic surface modification and functionalization, NiNPs can support multiple stimulus-responsive drug release mechanisms, further improving precision in controlled delivery applications (Farzin et al., 2020; Singh et al., 2023). NiNPs offer considerable benefits in precision cancer therapy through multidimensional delivery approaches, including the development of diverse magnetic nanocarriers, surface functionalization strategies, and the application of external magnetic fields for fine-tuned regulation, thereby achieving accurate *in vivo* drug delivery and controlled release (Peng et al., 2024). Notably, NiNPs can produce multifunctional, synergistic antitumor effects by integrating complementary therapeutic modalities, such as drug delivery, magnetic hyperthermia, photothermal therapy (PTT), and chemodynamic therapy, thus addressing several limitations associated with conventional chemotherapy (Mukherjee et al., 2020).

Although immunotherapy and targeted therapy have revolutionized cancer treatment, persistent challenges remain in addressing drug-resistant tumors, achieving deep penetration of solid tumors, and reducing off-target effects (Fan et al., 2023). NiNPs offer distinctive advantages by enabling the integration of multiple therapeutic modalities within a single platform, thereby supporting personalized treatment strategies tailored to specific tumor characteristics. The magnetic responsiveness of NiNPs facilitates real-time imaging and controlled drug release, aligning with the demands of precision medicine in modern oncology (Alirezaie Alavijeh et al., 2019; Ji et al., 2022). Moreover, unlike persistent metallic nanoparticles, certain nickel-based compounds, such as nickel selenide (NiSe), are biodegradable and can transform into excretable forms *in vivo*, providing enhanced biosafety. These attributes position NiNPs as next-generation therapeutic agents capable of addressing the shortcomings of current treatments and meeting the evolving needs of cancer therapy.

This review highlights recent developments in nickel-based nanoparticles for cancer treatment, including synthesis methods, targeted delivery techniques, and therapeutic applications. By critically examining the advantages and limitations of NiNPs, this review explores their capacity to overcome the delivery challenges inherent in traditional anticancer therapies and outlines promising directions for future research. It aims to establish a theoretical foundation and technical framework for the development of innovative, effective, and safe nickel nanoparticle platforms to advance precision oncology.

## 2 Nickel nanoparticles’ characteristics

### 2.1 Synthesis methods

NiNPs typically exhibit polymorphic and cubic crystalline structures, with particle sizes generally ranging from 10 to 100 nm. The synthesis methods for NiNPs are commonly categorized into three principal approaches: physical, chemical, and biological techniques (Bohra et al., 2024; Gürsoy et al., 2024). Physical synthesis methods utilize a top-down approach, wherein bulk nickel materials are reduced to nanoparticles through mechanical force, thermal energy, or electromagnetic radiation. These techniques offer operational simplicity and scalability for industrial-scale production; however, they often require substantial energy input (Narender et al., 2022; Shahidi et al., 2022). In contrast, chemical synthesis primarily follows bottom-up strategies, constructing nanostructures at the molecular level via chemical reactions—most notably reduction processes. Although these reactions are rapid and cost-effective, they pose risks of chemical contamination that may compromise the purity of the final product (Aali et al., 2021; Kim et al., 2023). Biological synthesis methods provide environmentally friendly alternatives by employing plant extracts, microorganisms, or other biological materials as both reducing and stabilizing agents (Sivagami and Asharani, 2022; Tailor et al., 2023; Alam et al., 2025). These green synthesis approaches offer significant environmental advantages, including reduced toxicity and cost-effectiveness; however, challenges remain regarding reaction yield, batch-to-batch consistency, and colloidal stability, which require further optimization (Jaji et al., 2020; e Silva et al., 2024).

### 2.2 Magnetic, catalytic, and optical characteristics

NiNPs possess outstanding magnetic properties and are classified as soft magnetic materials, characterized by high magnetic moments and saturation magnetization (Zarenezhad et al., 2022). These properties allow for precise manipulation using external magnetic fields, facilitating targeted drug delivery applications. Furthermore, the intrinsic chemical stability of NiNPs contributes to relatively low toxicity, establishing a reliable foundation for magnetic guidance and magnetic hyperthermia treatments (Vinod and Philip, 2022; Du et al., 2024). In terms of electrical characteristics, NiNPs exhibit excellent conductivity and low resistivity, making them highly suitable for electronic and sensing applications (Chereches and Minea, 2019). NiNPs also demonstrate significant catalytic activity, primarily due to their large specific surface area and unsaturated coordination sites on surface atoms. These active sites promote a range of chemical processes, including redox and electrocatalytic reactions. Notably, NiNPs can mimic enzymatic activity, such as that of peroxidase and catalase, enabling regulation of ROS within the tumor microenvironment and providing a mechanistic basis for CDT (Zhang et al., 2022; Dawson et al., 2023). Regarding optical behavior, NiNPs exhibit notable light absorption in the near-infrared (NIR) region, driven by surface plasmon resonance effects. This property allows NiNPs to efficiently convert absorbed light energy into heat, supporting PTT (Xiong et al., 2022). Importantly, their optical absorption characteristics can be optimized by tuning particle size, morphology, and surface chemistry, thereby enabling compatibility with various laser excitation wavelengths. This adjustability permits precise photothermal control and enhances therapeutic efficacy (Lei et al., 2019; Du et al., 2021a).

### 2.3 Surface modification and functionalization

Surface modification and functionalization are essential strategies for broadening the application scope of NiNPs and enhancing their biocompatibility. The surface of NiNPs contains numerous active sites, which allow for the attachment of functional molecules through various chemical bonding methods. Common strategies include polymer coating, biomolecule conjugation, and metal shell deposition (Ly et al., 2024). Among these, polymer coating is one of the most widely adopted approaches, employing polymers such as polyethylene glycol (PEG), polyvinylpyrrolidone (PVP), and polydopamine (PDA) to form protective layers. These coatings prevent nanoparticle aggregation and oxidation, prolong circulation time in the bloodstream, and reduce recognition by the reticuloendothelial system (Shi et al., 2021; Zhang et al., 2023).

Biomolecule conjugation improves the targeting ability of NiNPs by attaching recognition ligands, such as antibodies, aptamers, or peptides, to the nanoparticle surface, enabling active targeting of specific cells or tissues. Examples include folate for folate receptor targeting, RGD peptides for integrin targeting, and antibodies for epidermal growth factor receptor targeting, all of which bind selectively to overexpressed receptors on tumor cells (Qin et al., 2023a; Yin et al., 2023). Stimuli-responsive functionalization represents a critical method for designing intelligent nickel-based drug delivery systems. This involves incorporating molecules or polymers that respond to specific stimuli, such as pH, temperature, enzymes, light, or magnetic fields, to enable controlled drug release under defined conditions (Kargozar et al., 2022). Advanced functionalization strategies, such as nickel-substituted hydroxyapatite (Ni-HAp) and RGD-functionalized nanowires, significantly enhance interactions with biological tissues and improve drug delivery efficiency (Kesharwani et al., 2024; Asghar et al., 2025; Lorenzoni et al., 2025).

The excellent physicochemical characteristics and tunable surface chemistry of NiNPs provide a solid foundation for their diverse applications in cancer diagnosis and therapy (Inam et al., 2024). Through surface modification and functionalization strategies, NiNPs have transitioned from basic nanomaterials to clinically relevant therapeutic platforms. Variations in chemical composition and structural design among nickel-based nanomaterials result in substantial differences in their magnetic, optical, and catalytic properties. These property differences directly influence their therapeutic potential and suitability for specific cancer treatment modalities. To assess the functional characteristics and comparative advantages of different nickel-based systems, Table 1 presents a comprehensive analysis of principal nickel-based nanomaterials currently under investigation. The following section will explore how these properties are translated into effective cancer treatment strategies and highlight recent progress in practical applications.

**TABLE 1:** Functional characteristics comparison of nickel-based nanomaterials in cancer therapy.

Material	Magnetic properties	Optical properties	Catalytic properties	Photothermal efficiency	Chemodynamic efficiency	Biocompatibility	Primary therapeutic mechanism	Experimental models	Ref.
Nickel nanoparticles	Soft magnetic material with high saturation magnetization	Near-infrared absorption with surface plasmon resonance effects	Fenton-like catalytic activity, peroxidase-mimicking properties	Moderate, heating to 45°C within 5 min	SAR: 450 W/g, moderate ROS generation	Good with surface modification, requires polymer coating	Magnetic hyperthermia, magnetic field-guided targeting	HeLa cells, mouse tumor models	Ma et al. (2022)
Urchin-like Ni nanoparticles	Ferromagnetic with high saturation magnetization, rapid magnetic field response	Morphology-enhanced light scattering and absorption, effective NIR absorption	High surface area catalysis, needle structure enhances reaction sites	Good, morphology effect enhances photothermal conversion	Significantly enhanced, morphology increases catalytic sites	Good, stable performance *in vitro* and *in vivo*	Magneto-mechanical destruction (“Magnetic knife” technology)	4T1 breast cancer cells, BALB/c mice	Liu et al. (2025)
Nickel oxide nanoparticles	Antiferromagnetic with weak magnetic moment	UV-visible absorption, optical bandgap ∼3.6–4.0 eV	Excellent peroxidase-like activity, enzyme-mimicking catalysis	Low, mainly in the UV-visible region	Highly efficient, 85% drug release at pH 5.5	Moderate, requires surface functionalization	pH-responsive drug release, oxidative stress induction	A431 epidermal cancer cells, skin cancer models	Bano et al. (2016)
NiSe@PDA Nanocomposites	Paramagnetic, suitable for T1-weighted MRI contrast enhancement	Strong NIR-II absorption (1,064 nm), excellent optical properties	Controlled ion release, antioxidant enzyme activity	Excellent, photothermal conversion efficiency of 48.4%	Good, biodegradable characteristics	Excellent, selenium component enhances biocompatibility	NIR-II photothermal therapy combined with MRI-guided imaging	4T1 breast cancer models, MRI imaging validation	Hu et al. (2021)
Nickel ferrite nanoparticles	Ferrimagnetic with high coercivity, excellent magnetic heating performance	Plasmonic properties, near-infrared optical response	Catalase-like activity, dual enzyme properties	Good, enhanced by plasmonic effects	Moderate, synergistic dual enzyme activity	Good, high stability of ferrite structure	Magnetic hyperthermia combined with magnetic targeting	HeLa cells, magnetic hyperthermia experimental models	Rio et al. (2020)
Ni-doped cobalt ferrite	Optimized magnetic properties, enhanced magnetic response	Tunable optical bandgap, controllable absorption spectrum	Enhanced antioxidant activity, ROS regulation capability	Moderate, optimized photothermal performance after doping	Significant, ROS-mediated cytotoxicity	Good, stable after doping ratio optimization	Reactive oxygen species-mediated apoptosis	MCF-7 breast cancer cells	Sundram et al. (2022)
Nickel cobalt phosphide nanoparticles	Synergistic bimetallic magnetism, T1/T2 dual-modal MRI performance	Significant near-infrared absorption, excellent optical properties	Enhanced electrocatalytic activity, multi-site catalytic reactions	Significant, efficient conversion under NIR laser	Enhanced, electrocatalytic synergistic effects	Excellent, good stability of bimetallic phosphide	T1/T2 dual-modal MRI imaging combined with photothermal ablation	Tumor cell lines, *in vivo* imaging models	Lu et al. (2022)
Nickel phosphide quantum dots	Quantum-sized magnetic effects	Quantum-confined optical properties, excellent NIR response	Rich surface catalytic sites, high catalytic activity	Highly efficient, significantly enhanced by quantum effects	Highly efficient, synergistic catalytic mechanisms	Excellent, improved biocompatibility due to quantum dot size effects	Photothermal therapy synergistic with chemotherapy drugs	Liposome delivery systems, tumor models	Qian et al. (2022)
Ni-doped vanadium pentoxide	Doping-induced weak magnetism	Visible light response, bandgap tuned to visible region	Synergistic oxidative catalytic activity, PI3K/Akt pathway regulation	Moderate, visible light-activated photothermal effects	Significantly enhanced, pathway-specific inhibition effects	Moderate, requires concentration control	Apoptosis induction, signaling pathway inhibition	Skin cancer cells, *in vivo* safety assessment	Nivetha et al. (2024)
Ni-Ti oxide films	Magnetic responsiveness, suitable for magnetic field control	Stable near-infrared photothermal effects	Biocatalytic activity, promotes bone differentiation	Stable, excellent long-term photothermal stability	Moderate, stable catalytic activity in biological environment	Excellent, good biocompatibility of titanium-based materials	Dual function of bone tumor suppression and bone tissue regeneration	Bone tumor models, tissue engineering applications	Yao et al. (2022)

## 3 Applications of nickel nanoparticles in cancer treatment

### 3.1 Drug delivery

NiNPs enhance therapeutic efficacy by modulating the tumor microenvironment through multiple mechanisms. These nanoparticles can reprogram tumor-associated macrophages from the pro-tumor M2 phenotype to the anti-tumor M1 phenotype, while simultaneously increasing vascular permeability via controlled generation of ROS, thereby improving drug penetration and accumulation within tumor tissues (Miao et al., 2017; Sang et al., 2021b). Additionally, the catalase-like activity of NiNPs enables the decomposition of endogenous hydrogen peroxide into oxygen, alleviating tumor hypoxia. NiNPs also disrupt the dense extracellular matrix through magnetically driven mechanical forces, further enhancing therapeutic penetration. These regulatory effects on the tumor microenvironment form a strong foundation for diverse therapeutic applications involving NiNPs.

Due to their nanoscale size and surface functionalization, NiNPs can be preferentially taken up by cancer cells, allowing for precise drug delivery to tumor sites. Appropriately engineered NiNPs can achieve targeted delivery of anticancer agents (Du et al., 2021b). For example, Bano et al. developed nickel oxide (NiO) nanoparticles encapsulating doxorubicin (DOX) and functionalized with bovine serum albumin–folic acid (BSA-FA) complexes, which enabled controlled drug release under acidic tumor conditions (pH = 5.5) and red light stimulation (Bano et al., 2016).

Sharma et al. designed magnetic nickel nanowires modified with RGD peptides to enhance tumor cell internalization via integrin receptor-mediated targeting, leading to significantly increased drug accumulation in tumor tissues (Sharma et al., 2015). Further studies demonstrated that lipid-based NINPs with surface-chelated nickel ions enhanced targeting capability, achieving up to 90% internalization in epidermal cancer cells (A431) (Benhabbour et al., 2012). Karaca et al. introduced magnetic nickel nanomachines as drug carriers capable of targeted delivery via wireless control and stimuli-responsive drug release, offering novel insights for precision cancer therapy (Karaca et al., 2021). Additionally, magnetic nickel nanowires functionalized with RGD peptides have shown improved tumor cell uptake through integrin-mediated mechanisms (Qin et al., 2023a). Ramasamy et al. synthesized a magnetic nanocarrier composed of a β-cyclodextrin–folate–dextran polymer coating over nickel–zinc ferrite, which demonstrated efficient drug loading, sustained release, and enhanced cytotoxicity through folate receptor-mediated endocytosis (Ramasamy et al., 2018).

NiNP-based targeted drug delivery systems enable precise drug distribution, significantly reducing exposure to healthy cells while enhancing drug solubility, *in vivo* stability, and bioavailability, thus minimizing therapeutic side effects (Sanità et al., 2020). This technological advancement is driven by the synergistic use of multidimensional delivery strategies. These include the design of diverse magnetic nanocarriers, surface functionalization (e.g., PEGylation, DOX/PTX conjugation, and folic acid targeting) to enhance stability and specificity, and the precise regulation of nanoparticle migration and biodistribution via external magnetic fields (Sheikh et al., 2021). This integrated drug delivery platform combines targeting efficiency, safety, and therapeutic effectiveness, representing a new paradigm in precision cancer treatment and offering a viable approach to overcome the limitations of traditional chemotherapy.

### 3.2 Photothermal therapy

PTT is a therapeutic approach that ablates solid tumors through light-induced local hyperthermia generated by photothermal agents (Zhi et al., 2020). It has attracted significant interest in non-invasive cancer treatment due to its high therapeutic efficacy, limited adverse effects on surrounding healthy tissues, and high spatial and temporal resolution, which enables precise treatment control and minimizes damage to normal tissues (Zhang et al., 2021b; Wen et al., 2023). In particular, second near-infrared (NIR-II) lasers have demonstrated superior performance, offering deep tissue penetration, relatively low photon energy, and higher maximum permissible laser exposure limits (Yan et al., 2024; Cui et al., 2025).

The photothermal properties of NiNPs have been extensively investigated for cancer therapy. Upon NIR laser irradiation, aqueous dispersions of NiNPs rapidly elevate in temperature. At the molecular level, this process involves the absorption of photons by conduction electrons in metallic nickel, followed by electron–phonon coupling, which converts photon energy into lattice vibrations, producing heat. The resulting thermal energy initiates multiple cell death pathways, including protein denaturation, DNA damage, and activation of apoptotic cascades (Ahmed et al., 2020). Temperatures above 42°C impair cellular metabolism and induce heat shock protein expression, while those exceeding 50°C cause protein coagulation and immediate necrosis (Paściak et al., 2022; Abbas et al., 2023). This efficient photothermal conversion is attributed to NiNPs’ strong NIR absorption and minimal energy dissipation through radiation. *In vivo* experiments have confirmed the therapeutic potential of NiNP-based PTT (Xu et al., 2022; Oudjedi and Kirk, 2025). Hu et al. (2021) developed a multifunctional NIR-II-responsive nanoplatform, namely, nickel selenide@polydopamine nanocomposites (NiSe@PDA NCs) for dual-modal imaging-guided PTT. This material exhibited a photothermal conversion efficiency of 48.4% under NIR-II irradiation and served as a T1-weighted magnetic resonance imaging (MRI) contrast agent, enabling effective MRI-guided treatment of malignant tumors in both *in vitro* and *in vivo* models. Compared to conventional NiNPs, NiSe demonstrates superior photothermal performance due to its modified electronic band structure and enhanced biocompatibility, which also reduces the risk of toxic nickel ion release (Abbas et al., 2023). Zhou et al. (2014) reported that PEG-modified nickel carbide nanocrystals (Ni_3_C NCs) acted as efficient photothermal agents with strong NIR absorption and photothermal stability, achieving effective tumor cell ablation both *in vitro* and *in vivo*. Yao et al. (2022) designed a nickel nanoparticle-doped semiconductor film (Ni–Ti oxide), synthesized via *in situ* reduction of nickel–titanium layered double hydroxides. This material exhibited stable photothermal effects under NIR irradiation, prolonged stability in physiological environments, and supported both osteogenic differentiation and angiogenesis—features that make it suitable for the combined treatment of bone tumors and deep-seated malignancies.

**FIGURE 1:**
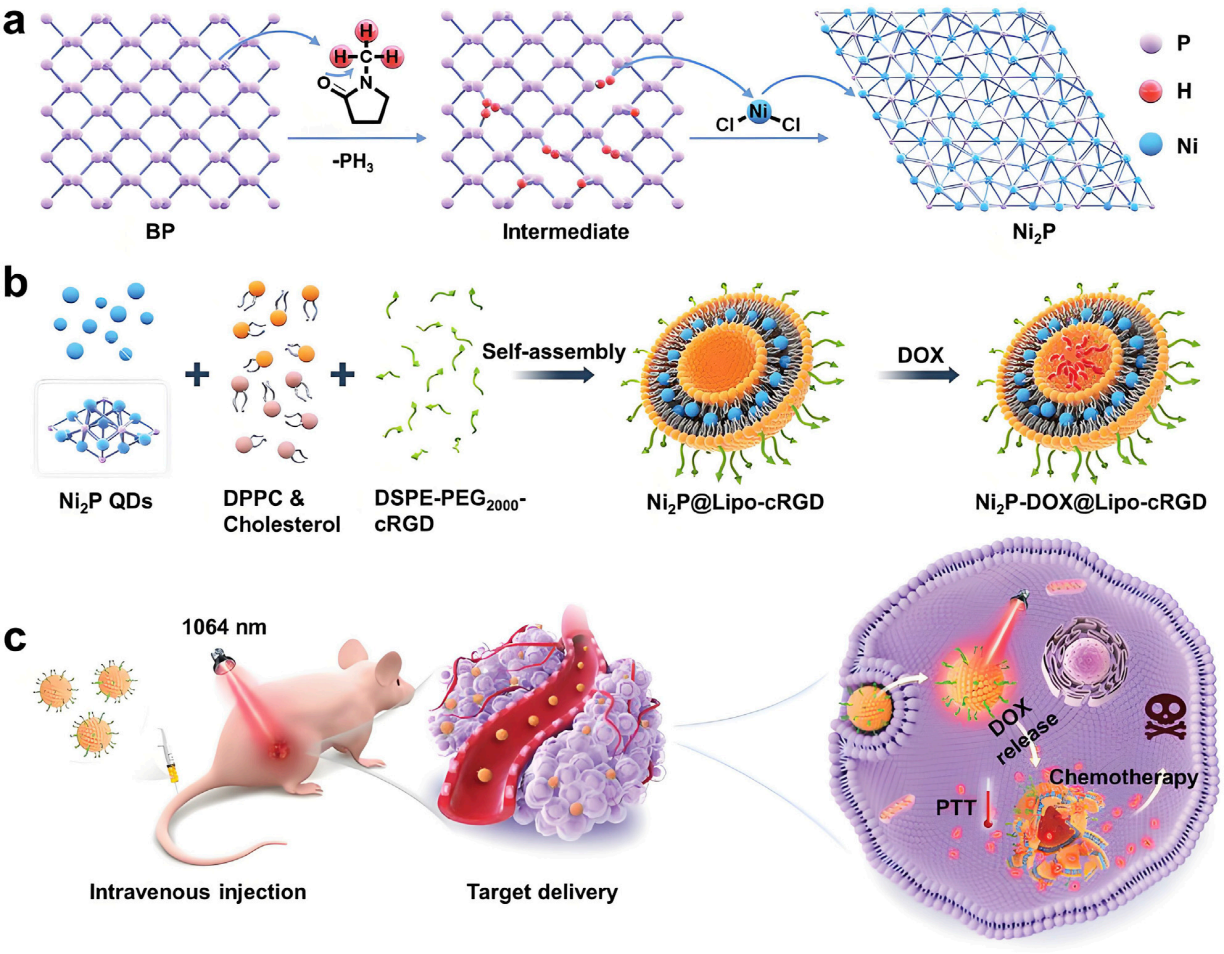
Schematic illustration of the development and therapeutic application of a nickel-based nanoplatform. The process includes: **(a)** topochemical synthesis of Ni_2_P quantum dots (QDs) from BPQD templates; **(b)** preparation of Ni_2_P-DOX@Lipo-cRGD through the incorporation of Ni_2_P QDs and doxorubicin (DOX) into liposomal structures; and **(c)** demonstration of the synergistic effects of Ni_2_P-DOX@Lipo-cRGD in achieving efficient tumor ablation through the combined mechanisms of PTT and chemotherapy. Reproduced with permission from Wu et al. (2024). Copyright (2024) Wiley-VCH GmbH.

For combination therapy, Wu et al. (2024) developed a liposomal nanoplatform (Ni_2_P-DOX@Lipo-cRGD), integrating Ni_2_P quantum dots and DOX into liposomal membranes and cores, respectively (Figure 1). *In vivo* studies demonstrated that the photothermal properties of Ni_2_P QDs enabled efficient tumor targeting, high biocompatibility, and complete tumor ablation through synergistic PTT and chemotherapy. In the context of gastric cancer treatment, Song et al. (2023) constructed a multifunctional nanoplatform (NNPIP NPs) that combined PTT with photodynamic therapy (PDT), leading to effective tumor reduction or eradication. These nanoplatforms also served as T1-weighted MRI contrast agents, supporting tumor diagnosis and preoperative staging while reducing toxicity and improving therapeutic selectivity. NiNPs hold substantial promise in PTT due to their excellent photothermal conversion efficiency, adjustable optical properties, and favorable biocompatibility. Through multifunctional integration, which encompasses imaging guidance, chemotherapeutic synergy, and targeted delivery, NiNPs not only enhance the efficacy of PTT but also present innovative treatment strategies for deep-seated and refractory tumors (Han and Choi, 2021; Alamdari et al., 2022).

### 3.3 Magnetic hyperthermia therapy

Magnetic hyperthermia is a targeted tumor treatment that selectively destroys cancer cells by generating localized heat within tumor tissues, thereby minimizing harm to adjacent healthy tissues (Vilas-Boas et al., 2020; Yu et al., 2022; Szwed et al., 2024). NiNPs, known for their high magnetic-to-thermal conversion efficiency, generate substantial heat via Néel and Brownian relaxation processes under alternating magnetic fields. Their specific absorption rate (SAR) can reach up to 450 W/g, which is sufficient to elevate tumor temperatures to therapeutic levels and induce cancer cell apoptosis (Ota and Takemura, 2019; Ahghari et al., 2020). The molecular mechanisms underlying magnetic hyperthermia involve two primary relaxation pathways. In Néel relaxation, the magnetic moment of individual nanoparticles flips relative to the crystal lattice in response to the external magnetic field, with the relaxation time determined by the energy barrier (KV/kBT), where *K* is the magnetic anisotropy constant, *V* is the particle volume, *k_B* is the Boltzmann constant, and *T* is the temperature (Ilg and Kröger, 2020). Brownian relaxation, by contrast, involves the physical rotation of the entire nanoparticle within its surrounding medium, with the relaxation time governed by hydrodynamic volume and fluid viscosity (Ota and Takemura, 2019; Torres et al., 2019). Both mechanisms convert magnetic energy into heat through frictional forces and reorientation of magnetic moments. The heat generated induces a range of cellular stress responses, including mitochondrial dysfunction, elevated ROS, and activation of temperature-sensitive ion channels (Shen et al., 2020). These stressors activate apoptotic signaling cascades involving caspase activation, cytochrome *c* release, and DNA fragmentation, resulting in targeted cancer cell death while sparing healthy tissue due to the confined nature of thermal induction (Ludwig et al., 2017; Clerc et al., 2018). Unlike photothermal therapies, which are limited by tissue light absorption and scattering, magnetic hyperthermia leverages magnetic fields with high tissue penetration, allowing effective treatment of deep-seated tumors. Additionally, it typically employs low-frequency, low-intensity magnetic fields that are non-harmful to human tissues, offering a safer and minimally invasive option for precision oncology (Sun et al., 2023).

Numerous studies support the therapeutic potential of nickel-based nanomaterials in magnetic hyperthermia. Rio et al. demonstrated that nickel ferrite (NiFe_2_O_4_) nanoparticles generate localized heat under alternating magnetic fields and possess both magnetic and plasmonic properties, enabling precise magnetic-guided positioning and synergistic thermal killing of cancer cells (Rio et al., 2020). Hopkins et al. (2017) designed nickel–gold core–shell nanowires (Ni–Au CSNWs) for radiofrequency-mediated thermal therapy. Remote activation of their paramagnetism using radiofrequency irradiation led to effective pancreatic tumor cell death, marked by nuclear shrinkage and fragmentation, thereby validating radiofrequency-induced thermal ablation. In another approach, Cabral et al. (2019) combined the internalization capabilities of boron nitride nanotubes (BNNTs) with the magnetic heating properties of NiFe_2_O_4_ nanoparticles to construct a robust hyperthermia platform. Following cellular uptake, this system successfully eradicated a majority of HeLa cancer cells in a single cycle using alternating current (AC) magnetic fields.

In summary, magnetic hyperthermia represents a promising cancer treatment modality enabled by the unique properties of NiNPs. Its advantages, including precise targeting, deep tissue penetration, and low systemic toxicity, make it particularly suitable for managing deep-seated and treatment-resistant tumors.

### 3.4 Chemodynamic therapy

CDT is an emerging tumor treatment strategy that has attracted considerable attention due to its non-invasive nature and minimal side effects. CDT based on Fenton or Fenton-like reactions generates highly toxic hydroxyl radicals (•OH) *in situ* within tumor tissues, inducing apoptosis and inhibiting tumor growth (Jia et al., 2022; Mohammed et al., 2022; Gao et al., 2023). At the molecular level, nickel-based nanoparticles catalyze the decomposition of endogenous hydrogen peroxide (H_2_O_2_) via Fenton-like reactions (Cao et al., 2022; Lin et al., 2023). This reaction involves the reduction of Ni^3+^ to Ni^2+^ in the presence of H_2_O_2_, accompanied by the generation of highly reactive •OH radicals. These radicals possess strong oxidative potential and non-selectively attack various biomolecules, including lipids, proteins, and nucleic acids (Koo et al., 2022). Lipid peroxidation disrupts membrane integrity, protein oxidation impairs enzymatic function and structure, and DNA damage leads to base modifications and strand breaks, thus collectively triggering apoptotic cell death pathways (Endale et al., 2023). The selectivity of CDT arises from the elevated H_2_O_2_ levels found in the tumor microenvironment relative to normal tissues, ensuring preferential ROS production within cancerous cells (Baghban et al., 2020; Yang et al., 2020; Chu et al., 2023). NiNPs have gained interest in this domain for their antioxidant modulation, Fenton-like catalytic activity, and integration within composite nanomaterials that enhance CDT efficacy, modulate the tumor microenvironment, and improve biosafety (Sang et al., 2021a). Their mechanisms of action include photocatalytic activity, enzyme-mimetic behavior, and biodegradability, offering new opportunities for precision, low-toxicity cancer therapy.

For example, nickel-based nanocomposites can induce apoptosis through catalytic generation of ROS. Nivetha et al. synthesized nickel-doped vanadium pentoxide (Ni@V_2_O_5_) nanocomposites, which inhibited skin cancer cell growth by inducing mitochondrial and nuclear damage, enhancing ROS generation, and activating caspase 9/3-mediated apoptotic signaling. Additionally, Ni@V_2_O_5_ suppressed the expression of oncoproteins, such as PI3K, Akt, and mTOR, supporting its potential as an anticancer agent (Nivetha et al., 2024). Similarly, Sundram et al. (2022) prepared nickel-doped cobalt ferrite (Ni-CFO) nanoparticles using coriander extract via a precipitation method. Among tested concentrations, 0.8% Ni-CFO exhibited strong magnetic and antioxidant properties. In MCF-7 breast cancer cells, 0.8% Ni-CFO induced apoptosis, inhibited cell adhesion and migration, and downregulated phosphorylated PI3K, Akt, and mTOR.

**FIGURE 2:**
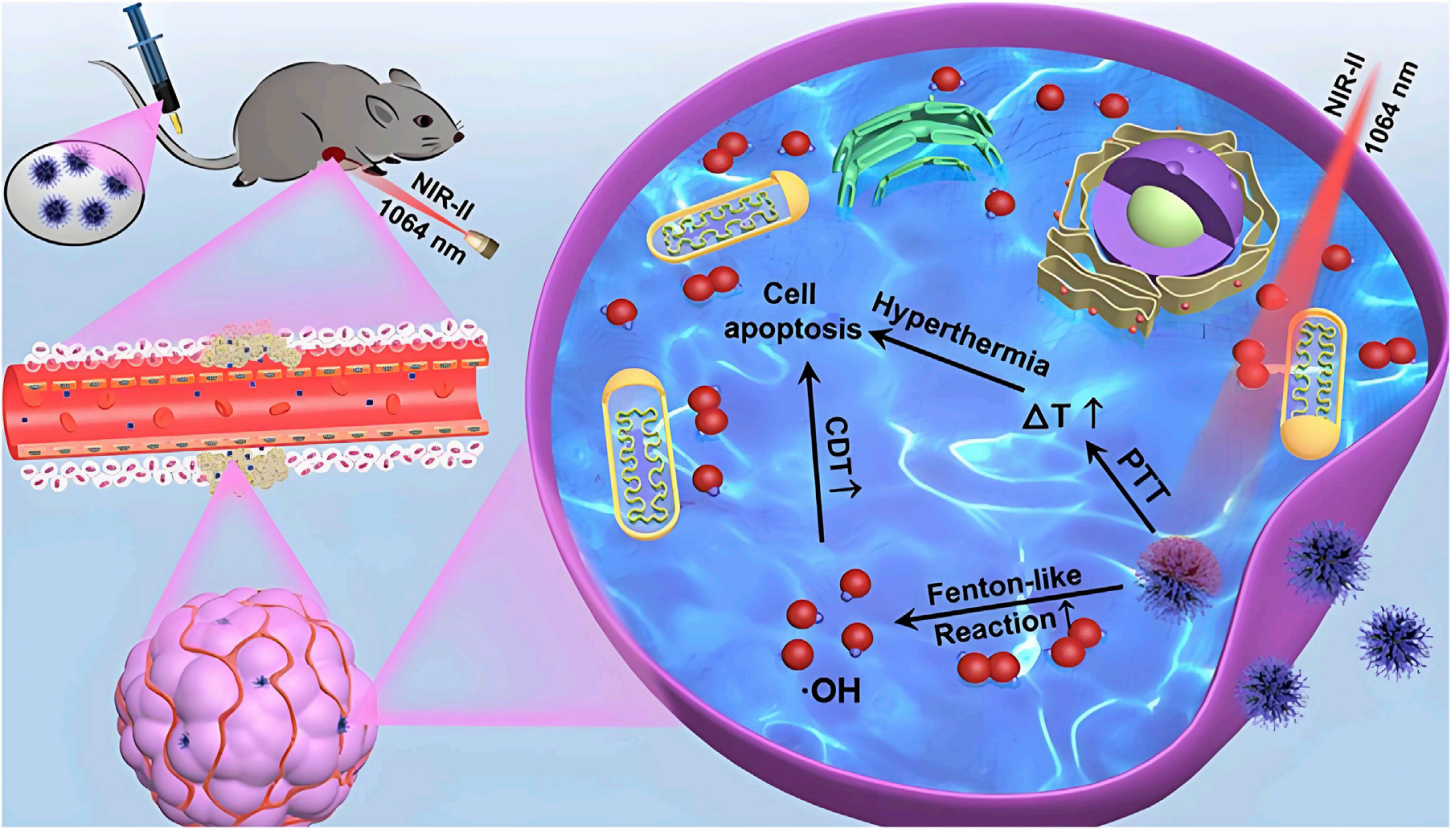
Schematic illustration of 9T-PUNNC nanoparticle-mediated photothermal-enhanced chemodynamic synergistic therapy. The diagram depicts how PEG-modified sea urchin-like nickel nanoclusters (PUNNCs) facilitate dual-mechanism cancer treatment. Under NIR-II irradiation, the structural features of PUNNCs enable excellent photothermal conversion and controlled Ni^2+^ ion release, enhancing chemodynamic therapy. This synergy results in demonstrated anticancer efficacy and biosafety in both *in vitro* and *in vivo* studies. Reproduced with permission from Qian et al. (2022). Copyright (2022) Ivyspring International Publisher.

Moreover, combining CDT with PTT has demonstrated synergistic therapeutic effects, as elevated temperatures not only facilitate thermal ablation but also accelerate Fenton reactions, enhancing ROS production (Zhang et al., 2020; Wang et al., 2021; Zhang et al., 2021a). Qian et al. (2022) developed PEG-modified sea urchin-like nickel nanoclusters (PUNNCs) for integrated NIR-II photothermal and chemodynamic therapy. The unique morphology of PUNNCs supported efficient photothermal conversion under NIR-II irradiation and enabled the controlled release of Ni^2+^ ions, thereby boosting CDT performance. Both *in vitro* and *in vivo* experiments confirmed the therapeutic efficacy and biosafety of PUNNCs, which effectively suppressed tumor growth and induced cancer cell death (Figure 2). In summary, CDT offers distinct advantages, including high tumor selectivity, minimal side effects, and no need for external energy input (Min et al., 2020). However, challenges remain in optimizing the catalytic efficiency and biocompatibility of nickel-based systems, as well as regulating H_2_O_2_ levels within the tumor microenvironment to maximize therapeutic outcomes.

### 3.5 Theranostic applications of nickel nanoparticles

NiNPs possess distinctive physicochemical properties that offer substantial potential not only for cancer treatment but also for diagnosis and imaging. Their exceptional magnetic and optical characteristics render them effective contrast agents for MRI and photoacoustic imaging, significantly improving tumor detection sensitivity and resolution compared to conventional imaging materials (Qian et al., 2022; Yu et al., 2017). Notably, their capacity to integrate diagnostic and therapeutic functions enables the realization of theranostic platforms, providing robust technical support for precision oncology.

Recent advancements in nickel nanoparticle-based theranostic systems have demonstrated remarkable multifunctionality. For example, Liu et al. (2018) developed a mesoporous nickel oxide (mNiO) nanoparticle system loaded with artemisinin (ART), capable of integrating T2-weighted MRI and NIR fluorescence imaging. This platform also functions as an efficient drug delivery system with controlled degradation and Ni^2+^ ion release under acidic tumor conditions. Additionally, it exhibits strong NIR absorption for PTT. Experimental data confirmed that this integrated strategy significantly enhanced antitumor efficacy in hypoxic tumor environments when compared to either free ART or PTT alone, highlighting the potential of natural product-based nanomedicine in cancer therapy. In another study, Lu et al. (2022) designed polyvinylpyrrolidone-coated bimetallic nickel–cobalt phosphide nanoparticles (NiCoP/PVP). Leveraging the complementary magnetic properties of nickel and cobalt, this system enabled dual-mode T1-and T2-weighted MRI, effectively addressing the limitations of single-modality imaging and enhancing diagnostic accuracy. NiCoP/PVP nanoparticles also exhibited strong NIR absorption and efficient photothermal conversion, making them effective agents for tumor photothermal ablation.

**FIGURE 3:**
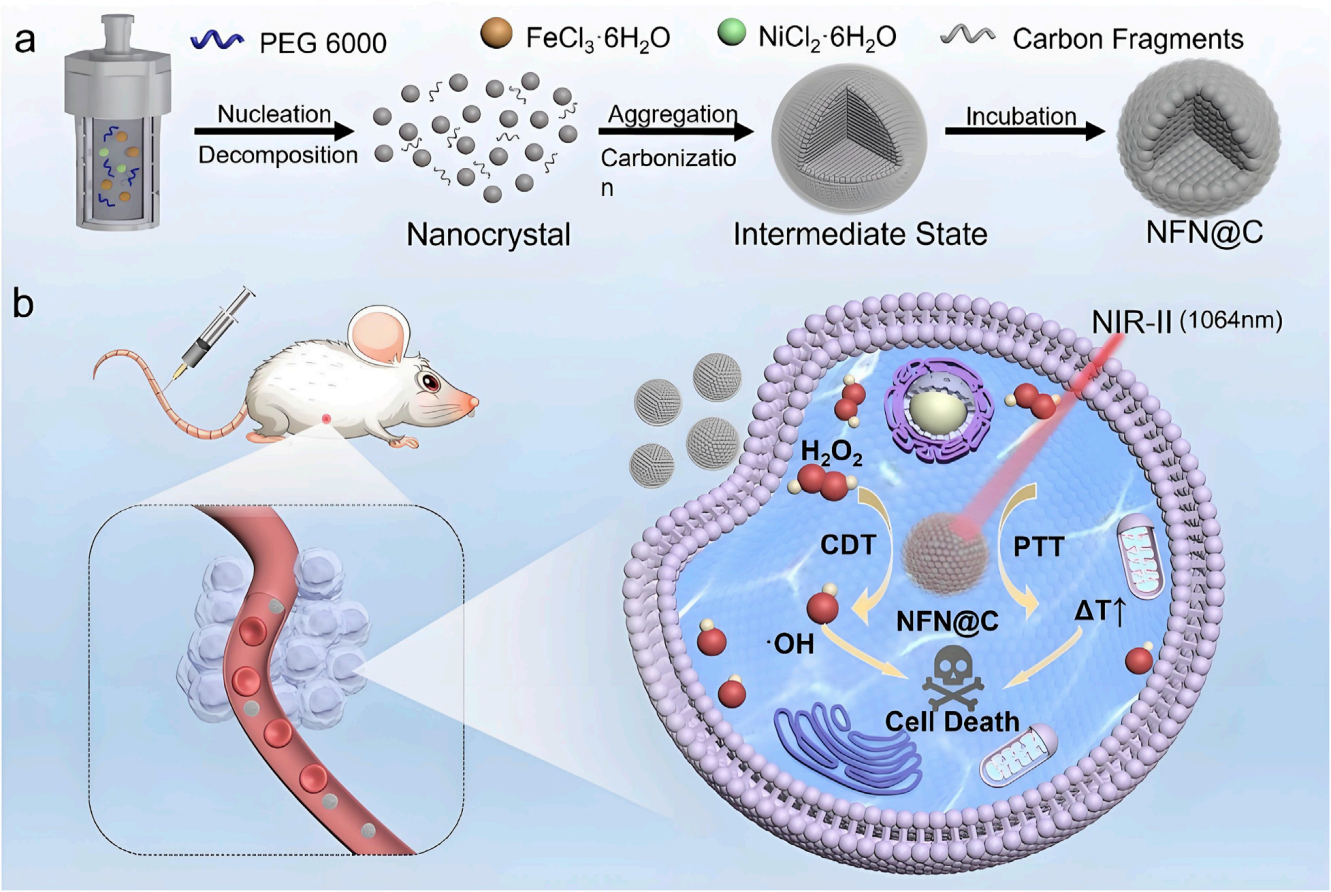
Schematic representation of the development and therapeutic mechanism of NFN@C nanocatalysts. The figure includes: **(a)** synthesis of carbon-coated nickel ferrite nanocatalysts (NFN@C), showing the incorporation of nickel into Fe_3_O_4_ crystal lattices; and **(b)** the mechanism by which NFN@C mediates photothermal-enhanced chemodynamic therapy. Nickel incorporation improves the electronic structure, increasing catalytic efficiency for H_2_O_2_ conversion into hydroxyl radicals within tumor microenvironments, while simultaneously enabling efficient photothermal conversion under NIR-II irradiation for synergistic therapeutic benefit. Reproduced with permission from Zhao et al. (2025). Copyright (2025) Wiley-VCH GmbH.

Significant progress has also been made in the development of catalytic nickel-based theranostic platforms. Liu et al. (2024) engineered nickel-based single-atom metal clusters (NSAMCs) that overcome several limitations of conventional iron-based agents in ferroptosis therapy. These clusters demonstrated excellent water solubility, colloidal stability, low toxicity, and selective tumor targeting. Their dual-enzyme mimetic activity synergistically induced cancer cell ferroptosis, significantly enhancing therapeutic efficacy. Another innovative approach involved the incorporation of nickel into Fe_3_O_4_ crystal lattices to produce carbon-coated nickel ferrite nanocatalysts (NFN@C). This structural modification optimized the electronic configuration of the catalyst, thereby enhancing its efficiency in catalyzing H_2_O_2_ into hydroxyl radicals (•OH) within tumor microenvironments. Electron paramagnetic resonance spectroscopy confirmed increased •OH production following nickel incorporation, indicating improved Fenton reaction efficiency due to electron density modulation. In addition to catalytic activity, NFN@C nanoparticles exhibited excellent NIR-II photothermal conversion capabilities, achieving synergistic effects between PTT and CDT that further improved antitumor outcomes (Zhao et al., 2025) (Figure 3).

### 3.6 Emerging applications of nickel nanoparticles

#### 3.6.1 Magneto-mechanical tumor destruction: the “magnetic knife” technology

**FIGURE 4:**
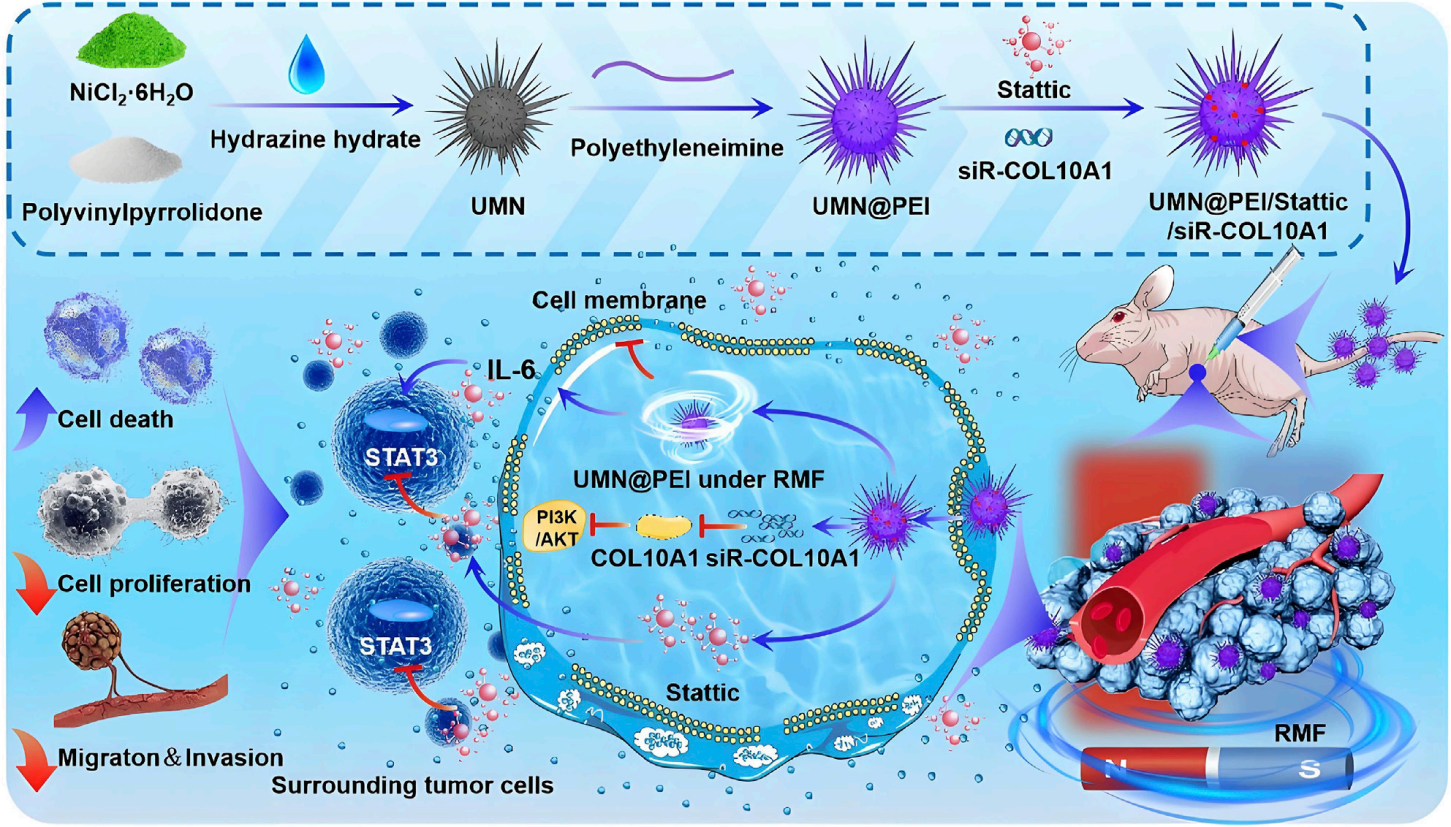
Schematic illustration of rotating magnetic field (RMF)-driven UMNP/St/si therapy for triple-negative breast cancer (TNBC). Urchin-like magnetic nanoparticles (UMNPs) are synthesized and loaded with the STAT3 inhibitor (Stattic) and COL10A1 siRNA to form UMNP/St/si complexes. When subjected to RMF, these nanoparticles disrupt cell membranes via magneto-mechanical forces, inhibit IL-6-induced STAT3 pathway activation, and suppress COL10A1 expression to inactivate the PI3K/AKT signaling pathway and remodel the extracellular matrix, ultimately reducing tumor growth. Reproduced with permission from Liu et al. (2025). Copyright (2025) Elsevier B.V.

The “magnetic knife” is an emerging anti-tumor strategy that destroys cancer cells through mechanical forces generated by magnetic nanoparticles under rotating magnetic fields (RMFs). These forces, resembling rotational stirring, physically disrupt tumor cells in a manner comparable to surgical excision (Wei and Wang, 2023; Zhao et al., 2023). Researchers have synthesized urchin-like nickel nanoparticles (UNNPs) via magnetic solvothermal methods, producing structures with high surface area and enhanced interaction with tumor cells. These nanoparticles exhibit high saturation magnetization and strong ferromagnetism, enabling rapid response to external magnetic fields. In both *in vitro* and *in vivo* models, UNNPs demonstrated effective tumor suppression and favorable biocompatibility. Their needle-like surface architecture significantly increased contact with cancer cells, resulting in elevated cell necrosis rates under RMF, and effectively inhibited breast cancer growth in mouse models (Qian et al., 2020). Liu et al. further developed a novel synthesis approach for urchin-like magnetic nanoparticles (UMNs) designed to treat triple-negative breast cancer (TNBC). These UMNs, prepared through a simplified solvothermal method, mechanically disrupted cell membranes under RMF, leading to increased tumor cell death. In addition, UMNs were loaded with a STAT3 inhibitor (Stattic) and COL10A1 siRNA to form UMNP/St/si complexes. The antitumor activity of this system under RMF was validated both *in vitro* and *in vivo* (Liu et al., 2025) (Figure 4). Kim et al. (2016) reported on magnetic nickel–gold-coated nanodisks functionalized with DNA aptamers, which effectively induced ascites cancer cell death under RMFs. This magnetically driven nanomechanical strategy holds strong potential for localized treatment of deep tumors, offering benefits such as simplicity, high efficiency, safety, and low cost (Lopez et al., 2022).

#### 3.6.2 Gene editing technology

Nickel-based platforms offer unique advantages for CRISPR-Cas9 delivery due to their magnetic targeting capabilities and controlled release mechanisms. The magnetic properties of NiNPs enable spatially precise delivery of gene editing components to tumor tissues. Their surface chemistry allows for the co-packaging of multiple CRISPR elements, including guide RNAs, Cas proteins, and donor DNA templates (Hryhorowicz et al., 2019; Rohiwal et al., 2020). Recent studies have shown that NiNPs can be functionalized with pH-responsive polymer coatings that protect the gene editing payload during circulation and facilitate release in acidic tumor environments (Duan et al., 2021). This design enhances tumor-specific accumulation while reducing off-target editing, which is a major limitation in conventional gene therapies. Furthermore, the photothermal properties of NiNPs can be harnessed to trigger localized release through controlled heating, enabling temporal regulation of gene editing activity.

#### 3.6.3 Immunotherapy

Recent advances suggest that NiNPs can be engineered for the precision delivery of immune checkpoint inhibitors. Their magnetic responsiveness enables targeted delivery of immunotherapeutic agents directly to tumor-associated immune cells (Walters et al., 2021). For example, NiNPs coated with anti-PD-L1 or anti-CTLA-4 antibodies can provide localized immune modulation while minimizing systemic immune-related adverse events (Kiaie et al., 2023). Magnetic guidance also facilitates accumulation in tumor-draining lymph nodes, which are key sites for initiating immune responses, thereby enhancing checkpoint blockade therapy effectiveness. Moreover, NiNPs have been employed to support chimeric antigen receptor T cell (CAR-T) therapy by magnetically labeling engineered T cells. This enables real-time tracking of CAR-T cell distribution and activity, improving therapeutic monitoring and potentially optimizing treatment outcomes (Qin et al., 2023b; Pfister et al., 2025).

## 4 Challenges in nickel nanoparticle applications

To comprehensively evaluate the clinical translation potential of NiNPs, Table 2 presents a comparative analysis of NiNPs and widely studied nanoparticle systems, such as iron oxide, gold, and copper-based platforms, based on key performance parameters. The therapeutic efficacy of NiNPs derives from their ability to convert various forms of energy (photonic, magnetic, and chemical) into localized cytotoxic effects via distinct molecular mechanisms.

**TABLE 2:** Comparative analysis of nickel nanoparticles with established nanoparticle systems for cancer therapy.

Classification Characteristics	Parameters	Nickel NPs	Iron oxide NPs	Gold NPs	Copper NPs
Magnetic properties	Saturation magnetization (emu/g)	55–60	60–90	Non-magnetic	Weak magnetic
	Magnetic behavior	Ferromagnetic	Superparamagnetic	-	Paramagnetic
	Targeting efficiency	High	High	-	Low
Photothermal properties	Conversion efficiency (%)	48–50	20–30	40–70	30–40
	Optimal NIR window	NIR-II (1,000–1,350 nm)	Limited	NIR-I (700–1,000 nm)	NIR-I
	Photostability	Good	Moderate	Excellent	Poor
Imaging capabilities	MRI contrast	T1/T2 dual-modal	T2-weighted	Limited	Limited
	CT contrast	Moderate	Low	Excellent	Moderate
	Photoacoustic imaging	Yes	Limited	Excellent	Moderate
Biocompatibility	Clinical approval status	Research stage	FDA approved	Research stage	Research stage
	Biodegradation	Controllable	Natural pathway	Minimal	Rapid but toxic
	Long-term safety	Requires optimization	Well-established	Concerns about retention	High toxicity risk
Economic factors	Raw material cost	Low-Moderate	Low	High	Low
	Synthesis complexity	Moderate	Low	High	Low
	Scale-up feasibility	Good	Excellent	Challenging	Good
Unique advantages	Key strengths	Multi-modal integration, NIR-II response	Clinical validation, biocompatibility	Excellent photothermal, imaging	Cost-effective
	Main limitations	Toxicity concerns	Limited photothermal	High cost, retention	Stability, toxicity
Ref.		Jaji et al. (2020), Wu and Kong (2020), Narender et al. (2022)	Zhu et al. (2018), Attia et al. (2022)	Sztandera et al. (2019), Hammami et al. (2021), Sani et al. (2021)	Ameh and Sayes (2019), Naz et al. (2020)

Despite these advantages, biosafety concerns represent major barriers to clinical application. Mo et al. (2024) demonstrated that nickel-containing nanoparticles exhibit dose-dependent cytotoxicity, genotoxicity, and carcinogenicity in lung tissues, with IC_50_ values ranging from 10 to 100 μg/mL in human lung epithelial cells. Ajdari and Ziaee Ghahnavieh (2014) conducted a comprehensive risk assessment showing that nickel-based nanomaterials tend to accumulate in the liver, spleen, and kidneys, with elimination half-lives exceeding 30 days in rodent models. Critical safety concerns include the kinetics of nickel ion release, dose–toxicity relationships, nanoparticle–immune system interactions, and degradation behavior under various physiological conditions.

NiNPs have also been shown to induce oxidative stress and inflammatory responses (Iftikhar et al., 2023). For example, NiO nanoparticles can cause apoptosis, necrosis, and IL-6/IL-8 secretion in lung epithelial cells (BEAS-2B, A549), and long-term exposure has been linked to chronic inflammation. Released nickel ions may interfere with intracellular calcium homeostasis and disrupt mitochondrial function. Although surface modification and delivery optimization can reduce toxicity, a trade-off exists between the thickness of protective coatings and the preservation of therapeutic functionality. Toxicity assessments employ both *in vitro* and *in vivo* models. Cell-based assays, including MTT, LDH release, and comet assays, have established dose–response relationships, typically observing cytotoxic effects at concentrations exceeding 50 μg/mL in various cancer cell lines (Mo et al., 2024). *In vivo* animal studies using rodent models have documented biodistribution patterns characterized by preferential accumulation in reticuloendothelial organs, with tissue nickel concentrations increasing by 10–15-fold relative to baseline following repeated administration (Abudayyak et al., 2020). Pharmacokinetic analyses indicate that surface-modified NiNPs exhibit biphasic elimination: an initial rapid clearance phase (t_1_/_2_ = 2–4 h) is followed by a prolonged retention phase (t_1_/_2_ = 15–30 days), suggesting a potential risk for long-term tissue accumulation (Di Bucchianico et al., 2018; Vallabani and Karlsson, 2022).

However, long-term risks, such as potential carcinogenicity and chronic organ dysfunction, remain inadequately characterized and require extended follow-up studies to establish definitive causal relationships. Moreover, inconsistencies in experimental protocols, nanoparticle properties, and administration regimens across studies complicate direct comparisons of safety profiles.

## 5 Summary and future prospects

Considering the potential and current limitations of NiNPs in cancer treatment, future research is likely to focus on the development of degradable NiNPs that retain therapeutic efficacy while enabling complete metabolic clearance, thereby reducing long-term toxicity risks.

Innovative strategies are emerging to address biocompatibility challenges through advanced surface engineering and controllable degradation mechanisms. Recent breakthroughs include the use of biomimetic cell membrane coatings derived from patient-specific cells, which offer immune evasion while maintaining magnetic and photothermal functionality (Jin et al., 2019). These coatings allow NiNPs to avoid immune detection without compromising their therapeutic properties. In addition, self-assembling peptide coatings have been developed to undergo conformational changes in response to tumor-specific enzymes, exposing targeting ligands exclusively within the tumor microenvironment (Liu et al., 2022).

The development of biodegradable NiNPs offers promising solutions to long-term safety concerns. These platforms employ controlled oxidation and complexation reactions to transform persistent nickel structures into excretable forms. One approach involves incorporating nickel into selenide structures that undergo predictable oxidation in biological environments, converting into water-soluble selenate compounds suitable for renal excretion (Menon et al., 2018). Such systems maintain therapeutic performance during treatment while ensuring complete elimination within defined timeframes. Compared to conventional NiNPs, NiSe offers several advantages: (1) reduced cytotoxicity due to a slower nickel ion release rate, which minimizes oxidative stress and cellular interference (Mikhailova, 2023); (2) enhanced biocompatibility attributed to selenium’s natural antioxidant properties; and (3) superior biodegradability via oxidation into water-soluble, physiologically eliminable selenates (Othman et al., 2023; Sowmya et al., 2024). Notably, this degradation process can release therapeutically beneficial selenium ions. Further, NiSe demonstrates improved photothermal and magnetic properties, with composite materials achieving photothermal conversion efficiencies approaching 50% while maintaining high biosafety standards (Abbas et al., 2023). Emerging monitoring technologies are also leveraging the magnetic properties of NiNPs for non-invasive tracking through advanced magnetic particle imaging. This enables clinicians to monitor nanoparticle biodistribution and clearance in real time with high spatial and temporal resolution, supporting adaptive treatment strategies based on patient-specific responses.

Future research will emphasize the design of NiNPs that respond to multiple physiological and biochemical signals in the tumor microenvironment to achieve precise drug release and therapeutic modulation. Exploiting the magnetic properties of NiNPs may also facilitate the development of real-time, non-invasive monitoring systems for *in vivo* tracking of nanoparticle distribution and degradation, laying the foundation for personalized treatment protocols. Simultaneously, exploring safer, more efficient, and environmentally sustainable synthesis methods will be essential to reduce production costs and enhance product consistency and quality.

In conclusion, while NiNPs hold considerable promise in cancer therapy, their clinical application remains in an exploratory phase. Future work must continue to address concerns related to biocompatibility and toxicity while advancing the design of safe, effective, and clinically translatable nickel-based nanotherapeutics.
